# CYP51C Y319H and H349R substitutions cannot confer azole resistance in *Aspergillus flavus* NRRL 3357-5

**DOI:** 10.1128/spectrum.00425-26

**Published:** 2026-06-09

**Authors:** Ya Bin Zhou, Alexey A. Grum-Grzhimaylo, Martin Meijer, Bart Kraak, Jos Houbraken

**Affiliations:** 1 Department of Dermatology, Peking University Third Hospital 66482, Beijing, China; 2 Westerdijk Fungal Biodiversity Institute 141042, Utrecht, the Netherlands; Mayo Foundation for Medical Education and Research, Rochester, Minnesota, USA

**Keywords:** *Aspergillus flavus*, azole resistance, *CYP51C*, site-directed mutagenesis, fitness cost

## Abstract

Azole antifungals inhibit the cytochrome P450-dependent lanosterol 14-α-demethylase (CYP51), an essential enzyme in the ergosterol biosynthetic pathway. While azole resistance mechanisms are well established in *Aspergillus fumigatus*, the molecular basis of resistance in *Aspergillus flavus* remains less defined. Previous studies identified nonsynonymous substitutions Y319H and H349R in CYP51C of azole-resistant *A. flavus* isolates, but their functional significance had not been experimentally confirmed. In this study, we used targeted gene replacement to introduce these mutations individually into a wild-type *A. flavus* background and assessed their impact on azole susceptibility and fitness. Sequence alignment revealed that residues Y319 and H349 are not conserved among fungal CYP51 (synonym “ERG11”) homologs. Both mutant strains exhibited the same susceptibility profiles to itraconazole, voriconazole, posaconazole, isavuconazole, echinocandins, and amphotericin B as the wild-type control. Moreover, colony growth and sporulation were comparable across all strains, indicating no measurable fitness cost associated with either substitution. These results indicate that CYP51C Y319H and H349R may not confer azole resistance or affect fungal fitness, underscoring the necessity of functional validation to distinguish neutral polymorphisms from true resistance determinants in *A. flavus*.

**IMPORTANCE:**

Azole resistance in *Aspergillus flavus* is an emerging clinical concern, yet its molecular basis remains poorly defined. This study demonstrates that two previously reported CYP51C substitutions (Y319H and H349R) do not confer azole resistance or affect fungal fitness when tested experimentally. Our findings highlight that the presence of mutations in resistant isolates does not necessarily imply causality. By emphasizing the need for functional validation, this work helps prevent misinterpretation of neutral polymorphisms as resistance markers and improves the accuracy of molecular diagnostics and resistance surveillance in *A. flavus*.

## INTRODUCTION

The lanosterol 14-α sterol demethylase enzyme (CYP51/ERG11) family is highly conserved across fungi and plays a pivotal role in ergosterol biosynthesis, a process essential for maintaining fungal cell membrane structure and function. In yeasts such as *Candida albicans* and *Saccharomyces cerevisiae*, this enzyme is encoded by a single gene, *erg11*, which catalyzes the oxidative demethylation of lanosterol, a key step in ergosterol production (1, 2). In contrast, filamentous fungi, including members of the genus *Aspergillus*, typically harbor multiple *CYP51* paralogs arising from gene duplication events, allowing potential functional specialization or redundancy (3, 4).

Azole antifungals, which inhibit CYP51, are the first-line agents for both treatment and prophylaxis of invasive aspergillosis (5). However, the increasing incidence of azole-resistant *Aspergillus* species poses a growing clinical and public health concern worldwide (6, 7). Resistance mechanisms have been most extensively studied in *Aspergillus fumigatus*, where point mutations and promoter tandem repeats in *cyp51A* are well-established drivers of azole resistance (8–10). By contrast, the molecular basis of azole resistance in *Aspergillus flavus*, which is the second most common cause of aspergillosis globally, remains incompletely understood (11, 12).

Unlike *A. fumigatus*, which possesses two *cyp51* paralogs (*cyp51A* and *cyp51B*), *A. flavus* contains three paralogs (*cyp51A*, *cyp51B*, and *cyp51C*) (13). Phylogenetic analysis suggests that *A. flavus* CYP51C shares greater amino acid identity with CYP51A (77%) than with CYP51B (60%), implying that *cyp51C* originated from an ancestral duplication of *cyp51A* (14, 15).

Nonsynonymous variants in CYP51C, such as Y319H and H349R, have been reported in azole-resistant *A. flavus* isolates (15, 16). Paul et al. (16) first identified a *cyp51C* T1025C mutation resulting in a Y319H substitution in a voriconazole-resistant *A. flavus* isolate and suggested a potential role in resistance. Similarly, an A1046G nucleotide change in *cyp51C* causing an H349R substitution was detected in another azole-resistant isolate (15). However, the functional roles of these substitutions in mediating azole resistance have not yet been validated experimentally.

Clarifying whether specific CYP51C substitutions confer resistance is of both diagnostic and clinical importance. *Aspergillus flavus* predominates in arid and high-temperature regions such as the Middle East, Africa, and Southeast Asia in immunocompromised individuals due to its thermotolerance and drought tolerance (17, 18). The emergence of azole-resistant *A. flavus* threatens current treatment strategies, particularly in resource-limited settings where antifungal susceptibility testing is not routinely performed. Identifying functionally relevant *cyp51C* mutations is therefore essential for resistance surveillance and molecular diagnostics.

Our recent work demonstrated that the CYP51A Y119F substitution directly contributes to azole resistance in *A. flavus* using a gene site mutation system (19). Employing the same experimental framework, we investigated whether naturally occurring CYP51C variants exert similar effects. Given the close evolutionary and structural relationship between CYP51A and CYP51C, this study aimed to functionally validate the roles of the *CYP51C* Y319H and H349R substitutions in azole resistance.

## MATERIALS AND METHODS

### Alignment of CYP51/ERG11 in different fungi

**Fig 1:**
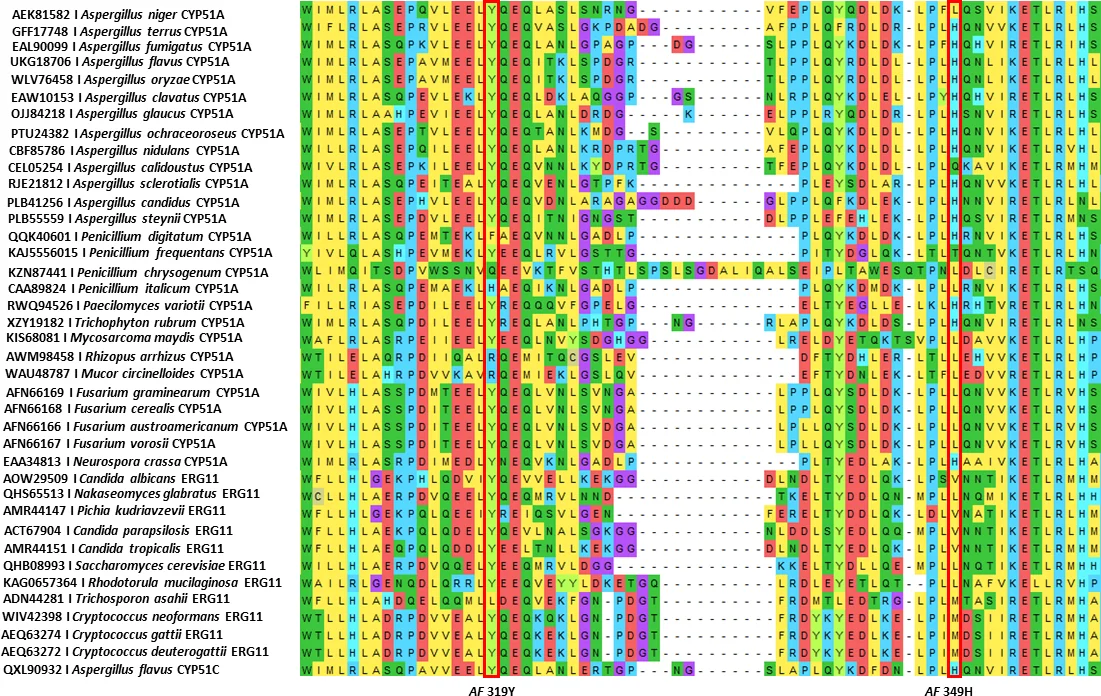
Alignment of positions 319Y and 349R in *A. flavus* CYP51C with CYP51A and ERG11.

A total of 39 fungal CYP51/ERG11 protein sequences were retrieved from GenBank and aligned using MEGA version 11. The tyrosine (Y319) and histidine (H349) residues of *A. flavus CYP51C*, along with the corresponding positions in the CYP51A or ERG11 homologs from other fungal species, were highlighted with red triangles (Fig. 1). The respective GenBank accession numbers for all sequences are provided in Fig. 1.

### Construction *cyp51C* point mutation cassettes

**Fig 2:**
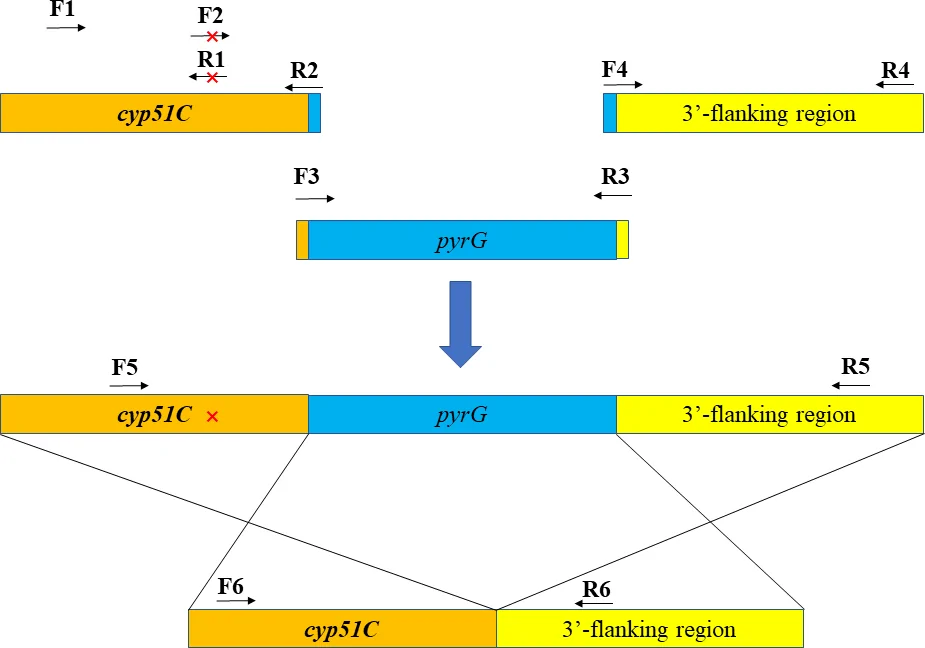
Strategy to construct *CYP51C* mutants.

The *cyp51*C point mutation cassettes were constructed using PCR-mediated overlap extension. Two overlapping fragments of the *cyp51C* gene were amplified from *A. flavus* NRRL 3357 genomic DNA using primer pairs F1/R1-C1025 or R1-G1046 and F2-C1025 or F2-G1046/R2, with the T1025C and A1046G mutations introduced through the primers R1-C1025/R1-G1046 and F2-C1025/F2-G1046, respectively. The resulting overlapping fragments were fused to generate a 1.5-kb PCR product carrying the desired *cyp51C* mutations. For construction of the wild-type control cassette, a *cyp51C* fragment was amplified from *A. flavus* NRRL 3357 genomic DNA using primer pair F1/R2. The *pyrG* selectable marker was amplified from plasmid *pFC330* (20) using primer pair F3/R3, and the 3′-flanking region of *cyp51C* was amplified from *A. flavus* NRRL 3357 genomic DNA using primer pair F4/R4. The complete mutant cassettes were assembled by fusing the mutated cyp51C fragments, *pyrG*, and the 3′-flanking sequence through a final amplification using primer pair F5/R5 (Fig. 2). A wild-type transformation cassette was constructed in parallel using the unmodified *cyp51C* sequence.

### Preparation of *A. flavus* protoplasts

Protoplasts of *A. flavus* were prepared as previously described (21), with minor modifications. Briefly, 50 mL of 1% glucose minimal medium supplemented with 5 mM uracil and 5 mM uridine was inoculated with 1 mL of *A. flavus* NRRL 3357-5 (a *pyrG* mutant of NRRL 3357) spore suspension (1 × 10⁷ spores/mL) in a 250 mL flask. The culture was incubated at 25°C with shaking at 200 rpm for 24 h. Mycelia were harvested by centrifugation at 4,000 × g for 10 min at 4°C and washed once with 15 mL JC buffer (0.8 M NaCl, 0.01 M sodium phosphate buffer) under the same conditions. The washed mycelia were then resuspended in 10 mL of filter-sterilized protoplasting solution containing 640 mg VinoTaste Pro (Novo Nordisk) dissolved in JC buffer and incubated for 3 h at 25°C with gentle agitation (100 rpm). Following incubation, the resulting protoplast suspension was filtered through two layers of Miracloth (Millipore) to remove undigested hyphae and centrifuged at 3,000 × g for 5 min at 4°C. The supernatant was discarded, and the protoplast pellet was gently resuspended in 1 mL STC buffer (1.2 M sorbitol, 10 mM Tris–HCl [pH 7.5], 10 mM CaCl₂). The concentration of the protoplast suspension was adjusted to 1 × 10⁷ protoplasts/mL using a hemocytometer.

### Transformation of *A. flavus* with *cyp51C* point mutation cassettes

Transformation of *A. flavus* NRRL 3357-5 protoplasts with *cyp51C* point mutation constructs was performed as previously described (21), with minor modifications. Briefly, 1 μg of the *cyp51C* point mutation cassette was added to 100 μL of freshly prepared protoplast suspension and incubated on ice for 5 min. Subsequently, 1 mL of filter-sterilized transformation solution containing 60% (wt/vol) polyethylene glycol 4000, 10 mM CaCl₂, and 0.8 M NaCl in 5 mM Tris–HCl (pH 7.5) was added to the mixture. The suspension was gently inverted to mix and incubated for 20 min at room temperature. The transformation mixture was then combined with 10 mL of pre-warmed (50°C) CZD top agar (Czapek–Dox liquid medium, 35 g/L; sorbitol, 182.2 g/L; (NH₄)₂SO₄, 1 g/L; Oxoid agar, 8 g/L), mixed gently by inversion, and poured onto plates containing 40 mL CZD bottom agar (Czapek–Dox liquid medium, 35 g/L; sorbitol, 182.2 g/L; (NH₄)₂SO₄, 1 g/L; Oxoid agar, 15 g/L).

**Fig 3:**
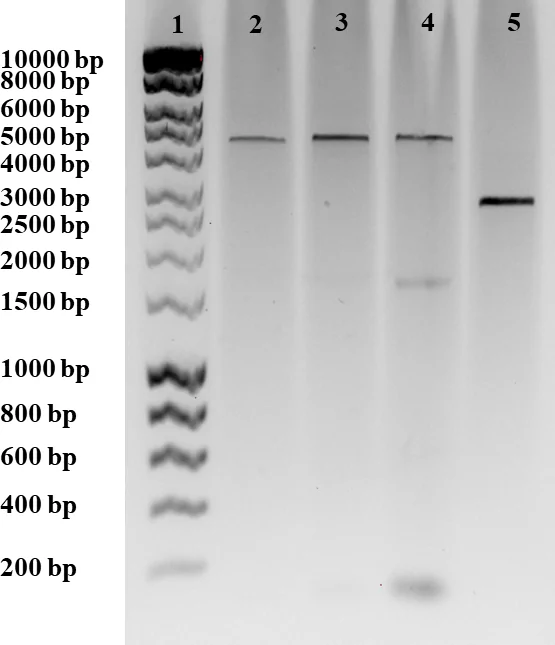
PCR verification of transformants. Lane 1, DNA marker; lane 2, DNA amplified from P51C^Y319H^; lane 3, DNA amplified from P51C^H349R^; lane 4, DNA amplified from P51C^WT^; lane 5, DNA amplified from NRRL 3357.

**Fig 4:**
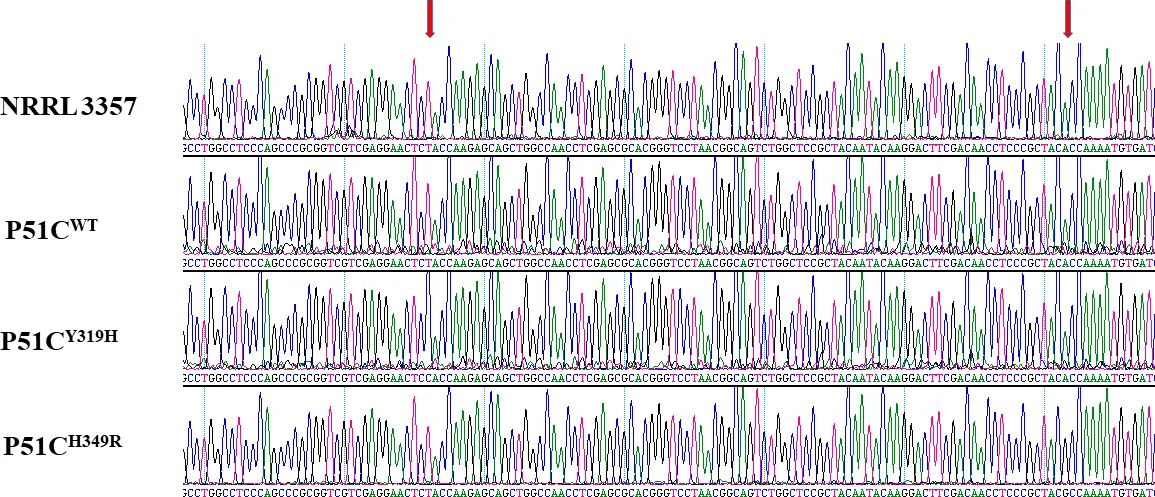
Sanger sequencing of transformants (arrowhead indicates mutation site). Transformant P51C^Y319H^ and P51C^H349R^ successfully incorporated the T1025C and A1046G mutation in the *cyp51C* gene, respectively.

Plates were incubated in the dark at 30°C for 3 days to allow colony development. Emerging transformants were subcultured onto fresh Czapek’s agar (CZA) plates lacking uracil and uridine for selection, and purified by two rounds of single-colony streaking on CZA. Genomic DNA was extracted from putative transformants and used as a PCR template for verification. Correctly integrated mutants were identified by amplification of the *cyp51C* gene using primer pair F6/R6 (Fig. 3), followed by Sanger sequencing (Fig. 4). The verified mutants containing the CYP51C Y319H and H349R substitutions, as well as the wild-type control, were designated P51C^Y319H^, P51C^H349R^, and P51C^WT^, respectively. All primers used in this study are listed in Table 1.

**TABLE 1:** Primers used in this study^*a*^

Name	Sequence (5′–3′)
F1	GCATGCTGAACAAGACTAGGCCGCCTCTGG
R1-C1025	*TTGGCCAGCTGCTCTTGGTGGAGTTCCTCGACGACCGCGG*
R1-G1046	*CTCTCGGATCACATTTTGGCGTAGCGGGAGGTTGTCGAAG*
F2-C1025	*CCGCGGTCGTCGAGGAACTCCACCAAGAGCAGCTGGCCAA*
F2-G1046	*CTTCGACAACCTCCCGCTACGCCAAAATGTGATCCGAGAG*
R2	*AACATTGGTCAATCACTGGT*TTATCCCGATTTTGCAGCCC
F3	*GGGCTGCAAAATCGGGATAA*ACCAGTGATTGACCAATGTT
R3	*TTGGTTCTCGAGATAATCGA*GCGGAAAACATGGCATTACA
F4	*TGTAATGCCATGTTTTCCGC*TCGATTATCTCGAGAACCAA
R4	GCAGGCGGAGGAAGCCAGAAAGAAGATGGT
F5	CCCCTTGACAACGCCAGTAT
R5	TGGTACAGCGGGTCCATCTA
F6	GACAGGCAGTGATACCACCA
R6	ACTGCACCCCTGGGATTTAT

^*a*^ The italic parts in the primers were the complementary tails to the adaptor primer and the underlined base in the primers was mutation site.

### Antifungal susceptibility testing

Antifungal susceptibility testing was performed using the broth microdilution reference method according to the Clinical and Laboratory Standards Institute (CLSI) M38-A3 guidelines (22). Susceptibility to itraconazole, voriconazole, posaconazole, isavuconazole, caspofungin, micafungin, anidulafungin, and amphotericin B was assessed. All antifungal powders were obtained from Merck (Darmstadt, Germany) and prepared according to CLSI recommendations. The final concentration ranges tested were 0.03–16 mg/L for itraconazole, voriconazole, posaconazole, isavuconazole, and amphotericin B, and 0.008–4 mg/L for caspofungin, micafungin, and anidulafungin.

Plates were incubated at 35°C for 48 h before reading. Minimum inhibitory concentrations (MICs) were defined as the lowest drug concentrations that completely inhibited visible growth. Minimum effective concentrations (MECs) for echinocandins were determined as the lowest concentrations that produced small, rounded, compact hyphal forms compared to the normal filamentous growth observed in the drug-free control wells. All assays were performed in triplicate on three independent days. *Aspergillus fumigatus* ATCC MYA-3626 and *Candida parapsilosis* ATCC 22019 were included as quality control strains.

### Morphological analysis

For growth assessment, strains were inoculated onto potato dextrose agar (PDA) plates using a 2 μL inoculum containing 1 × 10⁶ spores/mL, in triplicate. Plates were incubated at 30°C for 5 days, and colony diameters were measured daily using a ruler.

For sporulation analysis, conidia were harvested from 3-day-old PDA cultures incubated at 30°C by flooding the colony surface with sterile distilled water. The resulting spore suspension was serially diluted tenfold, and spore concentrations were determined using a hemocytometer under a light microscope. Each experiment was performed independently three times.

### Statistical analysis

Data analysis and graph generation were performed using GraphPad Prism version 8 (GraphPad Software, San Diego, CA, USA). Results are expressed as the mean ± standard deviation (SD). Statistical significance between two groups was evaluated using Student’s *t* test, and a *P* value of <0.05 was considered statistically significant.

## RESULTS

### CYP51C Y319 and H349 are not conserved amino acids in fungi

Sequence alignment of *A. flavus* CYP51C with CYP51 and ERG11 homologs from diverse fungal species revealed that the tyrosine residue at position 319 (Y319) and the histidine residue at position 349 (H349) are not conserved across fungi (Fig. 1). At these positions, Y319 is variably substituted by F, Q, H, R, or L, and H349 by Q, T, L, V, or M.

### CYP51C Y319H and H349R substitutions maintain susceptibility to azoles in *A. flavus*

Antifungal susceptibility testing showed that the *cyp51C* mutants exhibited identical susceptibility to itraconazole, voriconazole, posaconazole, isavuconazole, caspofungin, micafungin, anidulafungin, and amphotericin B compared with the wild-type strains (Table 2). This phenotype was consistently observed in the transformants P51C^Y319H^ and P51C^H349R^, relative to P51C^WT^ and NRRL 3357. These results demonstrate that the sole substitutions of CYP51C Y319H and H349R in *A. flavus* NRRL 3357-5 do not confer azole resistance under the investigated conditions.

**TABLE 2:** Antifungal susceptibilities of strains in this study^*a*^

Strains	MIC/MEC (mg/L)							
	ITC	VRC	POS	ISA	AMB	CAS	MCF	AND
P51C^Y319H^	1	0.5	0.125	0.5	1	0.06	≤0.008	≤0.008
P51C^H349R^	1	0.5	0.125	0.5	1	0.06	≤0.008	≤0.008
P51C^WT^	1	0.5	0.125	0.5	1	0.06	≤0.008	≤0.008
3357	1	0.5	0.125	0.5	1	0.06	≤0.008	≤0.008

^*a*^ ITC, itraconazole; VRC, voriconazole; POS, posaconazole; ISA, isavuconazole; AMB, amphotericin B; CAS, caspofungin; MCF, micafungin; AND, anidulafungin.

### CYP51C Y319H and H349R substitutions do not cause fitness cost in *A. flavus*

**Fig 5:**
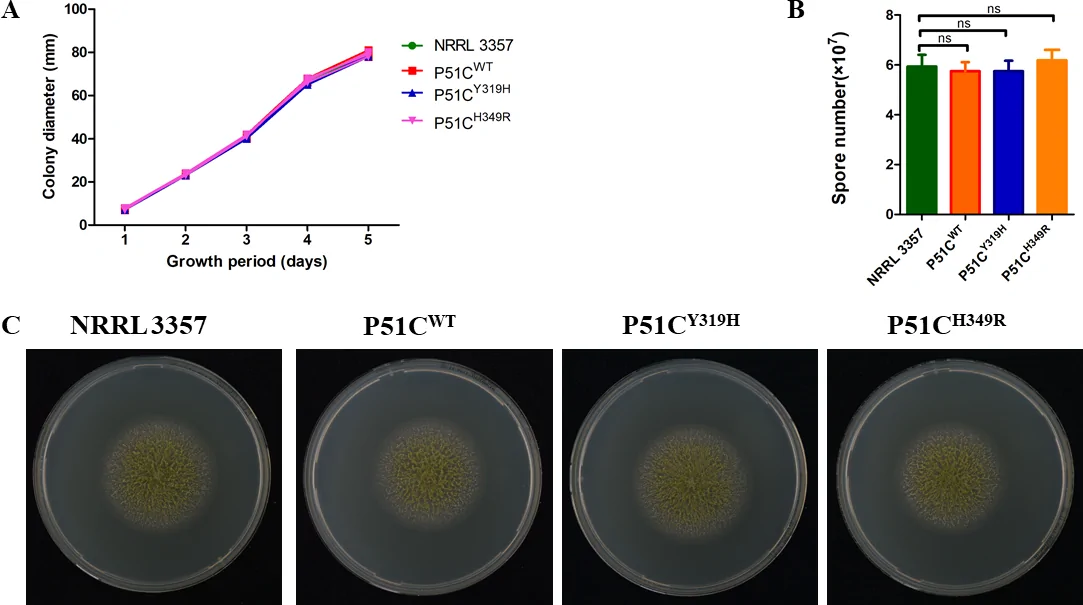
(**A**) Colony growth quantification of strains. (**B**) Comparison of spore numbers after 3 days of incubation at 30°C on PDA plates. (**C**) Colonies on PDA dishes after incubation at 30°C for 3 days. ns, not significant.

During five days of incubation on PDA, all strains (P51C^Y319H^, P51C^H349R^, P51C^WT^, and NRRL 3357) exhibited similar colony diameters at each time point (Fig. 5A). Sporulation was also comparable among the strains (Fig. 5B and C), indicating that the CYP51C Y319H and H349R substitutions do not impose a detectable fitness cost in *A. flavus* based on the results from the tested media.

## DISCUSSION

In this study, we evaluated two naturally occurring substitutions in CYP51C (Y319H and H349R) in *A. flavus* and found that neither mutation alone conferred azole resistance nor imposed a measurable fitness cost under the tested laboratory conditions. This finding helps clarify earlier reports suggesting possible associations between *cyp51C* variants and azole resistance phenotypes.

Sequence alignments of *A. flavus* CYP51C with CYP51A and *ERG11* homologs from diverse fungal species revealed that Y319 and H349 are not conserved residues across fungi. At these positions, a variety of amino acids (e.g., F, Q, H, R, and L at position 319; Q, T, L, V, and M at position 349) appear naturally, suggesting that substitutions at these positions may be tolerated and are unlikely to inherently destabilize enzyme structure or binding in a way that promotes resistance. The lack of conservation thus provides a rationale for our functional results. Similar variability in non-conserved residues has been observed across *Aspergillus* and *Candida s*pecies, where only mutations within conserved substrate recognition sites or the heme-binding region are typically associated with resistance (23–26). To the best of our knowledge, our manuscript is the first to attempt an alignment of *A. flavus* CYP51C with CYP51A or ERG11 from other fungal species. We believe this is because the *cyp51C* gene is present only in *A. flavus* and closely related species, while most other fungi lack this gene. Although CYP51C homologs have been reported in *Fusarium* species, these form a distinct phylogenetic subgroup and are therefore not directly comparable to *A. flavus* CYP51C (15). Based on our results, comparison of *A. flavus* CYP51C with CYP51A or ERG11 from other fungal species provides meaningful insights.

Our experimental approach—introducing specific *cyp51C* point mutations into a wild-type background—is the most reliable strategy for verifying the functional impact of amino acid substitutions. Indeed, functional genetic validation has been critical in confirming azole resistance mechanisms in *A. fumigatus* (23, 26–28) and, more recently, in *A. flavus* (12, 19). In contrast, reliance solely on molecular modeling or *in silico* docking studies cannot establish causality between specific mutations and resistance phenotypes. For example, Paul et al. (16) reported a Y319H substitution in *A. flavus* CYP51C in a voriconazole-resistant isolate and used molecular dynamics simulations to propose that the mutation increased protein flexibility and reduced drug binding. However, the study itself acknowledged that experimental confirmation—by introducing the Y319H mutation into a wild-type strain—was required to validate the hypothesis. In fact, the Y319H substitution was recently identified by Wang et al. (29) in an azole-susceptible strain. Our findings demonstrate that, in our laboratory strains, the substitutions Y319H and H349R alone do not alter azole susceptibility. The Y319H and H349R substitutions are unlikely to be associated with azole resistance. However, further validation is required, as this observation contradicts previous reports. For example, reverting the Y319H and H349R substitutions in clinically azole-resistant strains to assess whether azole susceptibility is restored would help clarify their role.

Our finding that the Y319H and H349R substitutions impose no detectable fitness cost—measured by colony diameter over 5 days on PDA and sporulation output—means these polymorphisms could persist in natural or clinical populations without a selective disadvantage. This is consistent with recent data showing that many resistance-associated *erg11* mutations in yeasts do not impose large fitness penalties (for example, deep mutational scanning in *Candida* showing only ~9% of resistant mutations caused significant fitness drops) (30). A similar phenomenon has been reported in *A. fumigatus*, where certain azole-resistant strains carrying *cyp51A* mutations exhibit minimal or no fitness cost (31, 32). The fitness costs observed in clinical azole-resistant isolates may result from additional mutations acquired during the development of azole resistance.

In our opinion, the current evidence linking CYP51C substitutions to azole resistance in *A. flavus* remains inconclusive and should be interpreted with caution. The Y319H and H349R substitutions were each reported only once in azole-resistant strains in the original studies and have not been consistently observed in other resistant isolates. The inference that these substitutions are associated with azole resistance appears to be based solely on their presence in single resistant strains. However, given the limited scope of existing studies, such conclusions may be premature. Importantly, the occurrence of a substitution in an azole-resistant strain does not preclude its presence in susceptible strains. This is exemplified by Y319H, which was subsequently identified in an azole-susceptible strain in a later study. Furthermore, CYP51C exhibits substantially higher polymorphism, with 38 reported amino acid substitutions, compared to CYP51A (13 substitutions) and CYP51B (four substitutions) in *A. flavus* (11). This elevated variability further complicates the interpretation of individual substitutions.

We therefore propose a more rigorous framework for assessing the potential association between CYP51C substitutions and azole resistance in *A. flavus*: (i) Epidemiological consistency: determine how frequently specific CYP51C substitutions occur across multiple azole-resistant strains. If the same substitution is consistently found in independent resistant isolates, this strengthens the likelihood of an association. In contrast, substitutions identified in single isolates should be interpreted cautiously. (ii) Comparative and structural context: align CYP51C from *A. flavus* with CYP51A or ERG11 from other fungal species. Substitutions located at conserved residues—particularly those already implicated in azole resistance in other fungi—are more likely to be functionally relevant. Substitutions outside conserved regions are less likely to contribute to resistance. (iii) Functional validation: introduce the substitutions into an azole-susceptible strain to assess whether they confer resistance. If the engineered strain exhibits reduced azole susceptibility, this supports a causal role. Conversely, if no effect is observed, the substitutions are unlikely to be involved in resistance. Additionally, reverting these substitutions in azole-resistant strains to evaluate whether susceptibility is restored would provide further confirmation.

## References

[1] Kelly SL, Kelly DE. 2013. Microbial cytochromes P450: biodiversity and biotechnology. Where do cytochromes P450 come from, what do they do and what can they do for us? Philos Trans R Soc Lond B Biol Sci 368:20120476. doi:10.1098/rstb.2012.0476 23297358 PMC3538425

[2] Sanglard D. 2016. Emerging threats in antifungal-resistant fungal pathogens. Front Med 3:11. doi:10.3389/fmed.2016.00011 PMC4791369 27014694

[3] Becher R, Wirsel SGR. 2012. Fungal cytochrome P450 sterol 14α-demethylase (CYP51) and azole resistance in plant and human pathogens. Appl Microbiol Biotechnol 95:825–840. doi:10.1007/s00253-012-4195-9 22684327

[4] Alcazar-Fuoli L, Mellado E. 2014. Current status of antifungal resistance and its impact on clinical practice. Br J Haematol 166:471–484. doi:10.1111/bjh.12896 24749533

[5] Douglas AP, Smibert OC, Bajel A, Halliday CL, Lavee O, McMullan B, Yong MK, van Hal SJ, Chen SC-A, Australasian Antifungal Guidelines Steering Committee. 2021. Consensus guidelines for the diagnosis and management of invasive aspergillosis, 2021. Intern Med J 51 Suppl 7:143–176. doi:10.1111/imj.15591 34937136

[6] Lockhart SR, Chowdhary A, Gold JAW. 2023. The rapid emergence of antifungal-resistant human-pathogenic fungi. Nat Rev Microbiol 21:818–832. doi:10.1038/s41579-023-00960-9 37648790 PMC10859884

[7] Vermeulen E, Lagrou K, Verweij PE. 2013. Azole resistance in *Aspergillus fumigatus*: a growing public health concern. Curr Opin Infect Dis 26:493–500. doi:10.1097/QCO.0000000000000005 24126719

[8] Wiederhold NP, Patterson TF. 2015. Emergence of azole resistance in *Aspergillus*. Semin Respir Crit Care Med 36:673–680. doi:10.1055/s-0035-1562894 26398534

[9] Zubovskaia A. 2025. Azole-resistant *Aspergillus fumigatus*: epidemiology, diagnosis, and treatment considerations. J Fungi 11:731. doi:10.3390/jof11100731 PMC12565632 41149921

[10] Sen P, Vijay M, Singh S, Hameed S, Vijayaraghavan P. 2022. Understanding the environmental drivers of clinical azole resistance in *Aspergillus* species. Drug Target Insights 16:25–35. doi:10.33393/dti.2022.2476 36458152 PMC9685629

[11] Djenontin E, Lavergne RA, Morio F, Dannaoui E. 2025. Antifungal resistance in non‐fumigatus *Aspergillus* species. Mycoses 68:e70051. doi:10.1111/myc.70051 40219727 PMC11992613

[12] Ukai Y, Kuroiwa M, Kurihara N, Naruse H, Homma T, Maki H, Naito A. 2018. Contributions of *yap1* mutation and subsequent *atrF* upregulation to voriconazole resistance in *Aspergillus flavus*. Antimicrob Agents Chemother 62:e01216-18. doi:10.1128/AAC.01216-18 30126960 PMC6201102

[13] De Francesco MA. 2023. Drug-resistant *Aspergillus* spp.: a literature review of its resistance mechanisms and its prevalence in Europe. Pathogens 12:1305. doi:10.3390/pathogens12111305 38003770 PMC10674884

[14] Hawkins NJ, Cools HJ, Sierotzki H, Shaw MW, Knogge W, Kelly SL, Kelly DE, Fraaije BA. 2014. Paralog re-emergence: a novel, historically contingent mechanism in the evolution of antimicrobial resistance. Mol Biol Evol 31:1793–1802. doi:10.1093/molbev/msu134 24732957 PMC4069618

[15] Lucio J, Gonzalez-Jimenez I, Rivero-Menendez O, Alastruey-Izquierdo A, Pelaez T, Alcazar-Fuoli L, Mellado E. 2020. Point mutations in the 14-α sterol demethylase Cyp51A or Cyp51C could contribute to azole resistance in *Aspergillus flavus*. Genes (Basel) 11:1217. doi:10.3390/genes11101217 33080784 PMC7602989

[16] Paul RA, Rudramurthy SM, Meis JF, Mouton JW, Chakrabarti A. 2015. A novel Y319H substitution in CYP51C associated with azole resistance in *Aspergillus flavus*. Antimicrob Agents Chemother 59:6615–6619. doi:10.1128/AAC.00637-15 26248359 PMC4576050

[17] Rudramurthy SM, Paul RA, Chakrabarti A, Mouton JW, Meis JF. 2019. Invasive aspergillosis by *Aspergillus flavus*: epidemiology, diagnosis, antifungal resistance, and management. J Fungi 5:55. doi:10.3390/jof5030055 PMC6787648 31266196

[18] Van Der Linden JWM, Warris A, Verweij PE. 2011. *Aspergillus* species intrinsically resistant to antifungal agents. Med Mycol 49 Suppl 1:S82–S89. doi:10.3109/13693786.2010.499916 20662634

[19] Zhou Y, Wang Y, Grum-Grzhimaylo AA, Meijer M, Kraak B, Li Z, Houbraken J. 2025. Contribution of the CYP51A Y119F mutation to azole resistance in *Aspergillus flavus*. J Fungi 11:798. doi:10.3390/jof11110798 PMC12653524 41295178

[20] Nødvig CS, Nielsen JB, Kogle ME, Mortensen UH. 2015. A CRISPR-Cas9 system for genetic engineering of filamentous fungi. PLoS One 10:e0133085. doi:10.1371/journal.pone.0133085 26177455 PMC4503723

[21] Choupannejad R, Sharifnabi B, Collemare J, Gholami J, Mehrabi R. 2025. The candidate transcription factors PnAtfA, PnCrz1, and PnVf19 contribute to fungal morphogenesis, abiotic stress tolerance, and pathogenicity in the wheat pathogen *Parastagonospora nodorum*. Fungal Biol 129:101565. doi:10.1016/j.funbio.2025.101565 40222766

[22] The Clinical and Laboratory Standards Institute. 2017. Reference method for broth dilution antifungal susceptibility testing of filamentous fungi-third edition: approved standard M38-A3. CLSI, Wayne, PA.

[23] Snelders E, Camps SMT, Karawajczyk A, Rijs AJMM, Zoll J, Verweij PE, Melchers WJG. 2015. Genotype-phenotype complexity of the TR46/Y121F/T289A *cyp51A* azole resistance mechanism in *Aspergillus fumigatus*. Fungal Genet Biol 82:129–135. doi:10.1016/j.fgb.2015.06.001 26092193

[24] Sanguinetti M, Posteraro B, Lass-Flörl C. 2015. Antifungal drug resistance among *Candida* species: mechanisms and clinical impact. Mycoses 58 Suppl 2:2–13. doi:10.1111/myc.12330 26033251

[25] Feng W, Yang J, Xi Z, Qiao Z, Lv Y, Wang Y, Ma Y, Wang Y, Cen W. 2017. Mutations and/or overexpressions of *ERG4* and *ERG11* genes in clinical azoles-resistant isolates of *Candida albicans*. Microb Drug Resist 23:563–570. doi:10.1089/mdr.2016.0095 27976986

[26] Krishnan Natesan S, Wu W, Cutright JL, Chandrasekar PH. 2012. *In vitro*-*in vivo* correlation of voriconazole resistance due to G448S mutation (*cyp51A* gene) in *Aspergillus fumigatus*. Diagn Microbiol Infect Dis 74:272–277. doi:10.1016/j.diagmicrobio.2012.06.030 22897872

[27] Camps SMT, van der Linden JWM, Li Y, Kuijper EJ, van Dissel JT, Verweij PE, Melchers WJG. 2012. Rapid induction of multiple resistance mechanisms in *Aspergillus fumigatus* during azole therapy: a case study and review of the literature. Antimicrob Agents Chemother 56:10–16. doi:10.1128/AAC.05088-11 22005994 PMC3256077

[28] Snelders E, Karawajczyk A, Verhoeven RJA, Venselaar H, Schaftenaar G, Verweij PE, Melchers WJG. 2011. The structure-function relationship of the *Aspergillus fumigatus cyp51A* L98H conversion by site-directed mutagenesis: the mechanism of L98H azole resistance. Fungal Genet Biol 48:1062–1070. doi:10.1016/j.fgb.2011.08.002 21907818

[29] Wang H-C, Hsieh M-I, Choi P-C, Wu W-L, Wu C-J, TSARM Hospitals. 2023. Species distribution and antifungal susceptibility of clinical *Aspergillus* isolates: a multicentre study in Taiwan, 2016-2020. Mycoses 66:711–722. doi:10.1111/myc.13593 37186489

[30] Bédard C, Gagnon-Arsenault I, Boisvert J, Plante S, Dubé AK, Pageau A, Fijarczyk A, Sharma J, Maroc L, Shapiro RS, Landry CR. 2024. Most azole resistance mutations in the *Candida albicans* drug target confer cross-resistance without intrinsic fitness cost. Nat Microbiol 9:3025–3040. doi:10.1038/s41564-024-01819-2 39379635

[31] Valsecchi I, Mellado E, Beau R, Raj S, Latgé JP. 2015. Fitness studies of azole-resistant strains of *Aspergillus fumigatus*. Antimicrob Agents Chemother 59:7866–7869. doi:10.1128/AAC.01594-15 26416854 PMC4649223

[32] Chen S, Zhu G, Lin H, Guo J, Deng S, Wu W, Goldman GH, Lu L, Zhang Y. 2024. Variability in competitive fitness among environmental and clinical azole-resistant *Aspergillus fumigatus* isolates. mBio 15:e0026324. doi:10.1128/mbio.00263-24 38407058 PMC11005360

